# Targeting the Intracellular Environment in Cystic Fibrosis: Restoring Autophagy as a Novel Strategy to Circumvent the CFTR Defect

**DOI:** 10.3389/fphar.2013.00001

**Published:** 2013-01-21

**Authors:** Valeria Rachela Villella, Speranza Esposito, Emanuela M. Bruscia, Maria Chiara Maiuri, Valeria Raia, Guido Kroemer, Luigi Maiuri

**Affiliations:** ^1^European Institute for Research in Cystic Fibrosis, San Raffaele Scientific InstituteMilan, Italy; ^2^Department of Pediatrics, Yale University School of MedicineNew Haven, CT, USA; ^3^Department of Experimental Pharmacology, Federico II UniversityNaples, Italy; ^4^INSERM U848Villejuif, France; ^5^Cystic Fibrosis Unit, Department of Pediatrics, Federico II UniversityNaples, Italy; ^6^Université Paris DescartesParis, France; ^7^Metabolomics Platform, Institut Gustave RoussyVillejuif, France; ^8^Centre de Recherche des CordeliersParis, France; ^9^PTle de Biologie, HTpital Européen Georges Pompidou, Assistance Publique – HTpitaux de ParisParis, France; ^10^Institute of Pediatrics, University of FoggiaFoggia, Italy

**Keywords:** cystic fibrosis, CFTR, proteostasis regulators, autophagy, BECN1

## Abstract

Cystic fibrosis (CF) patients harboring the most common deletion mutation of the CF transmembrane conductance regulator (CFTR), F508del, are poor responders to potentiators of CFTR channel activity which can be used to treat a small subset of CF patients who genetically carry plasma membrane (PM)-resident CFTR mutants. The misfolded F508del-CFTR protein is unstable in the PM even if rescued by pharmacological agents that prevent its intracellular retention and degradation. CF is a conformational disease in which defective CFTR induces an impressive derangement of general proteostasis resulting from disabled autophagy. In this review, we discuss how rescuing Beclin 1 (BECN1), a major player of autophagosome formation, either by means of direct gene transfer or indirectly by administration of proteostasis regulators, could stabilize F508del-CFTR at the PM. We focus on the relationship between the improvement of peripheral proteostasis and CFTR PM stability in F508del-CFTR homozygous bronchial epithelia or mouse lungs. Moreover, this article reviews recent pre-clinical evidence indicating that targeting the intracellular environment surrounding the misfolded mutant CFTR instead of protein itself could constitute an attractive therapeutic option to sensitize patients carrying the F508del-CFTR mutation to the beneficial action of CFTR potentiators on lung inflammation.

## Introduction

The proteostasis network ensures intracellular homeostasis in spite of genetic or epigenetic changes in protein conformation, extracellular stress, or aging-associated perturbations (Balch et al., 2008; Hutt et al., 2009; Powers et al., 2009; Gidalevitz et al., 2010; Hutt and Balch, 2010; Roth and Balch, 2011). The accumulation of misfolded/modified proteins due to mutations or due to the aging-related decline of proteostasis contributes to several human conformational diseases including neurodegenerative disorders and type II diabetes (Balch et al., 2008; Powers et al., 2009; Gidalevitz et al., 2010; Roth and Balch, 2011).

Cystic fibrosis (CF), the most common life-threatening genetic disease among Caucasians, constitutes the quintessential example of a “conformational disease” (Balch et al., 2011; Okiyoneda et al., 2011). CF is caused by mutations of the CF transmembrane conductance regulator (CFTR) gene that encodes a cAMP-regulated chloride channel primarily located at the apical membrane of epithelial cells (Quinton, 1999; Welsh et al., 2001; Park et al., 2010). Although more than1800 different mutations have been identified, one single deletion of phenylalanine at position 508 (F508del-CFTR), occurs in about 70–90% of CF patients in Northern Europe and North America (Bobadilla et al., 2002). F508del-CFTR protein can still retain a partial chloride channel activity if rescued at the epithelial surface. However, due to its misfold, F508del-CFTR does not reach the plasma membrane (PM) and is prematurely degraded, thus provoking local inflammation, increased susceptibility to respiratory bacterial infections, and progressive pulmonary and digestive insufficiency (O’Sullivan and Freedman, 2009; Ratjen, 2009).

The birth prevalence of CF is estimated to be one in 3500–4500, with 200-300 new cases each year in Europe. The typical form of CF is diagnosed during early childhood and is characterized by recurrent pulmonary infections, pancreatic insufficiency, and elevated chloride concentrations in sweat. Although CF is a systemic disease, the main cause of death is persistent and untreatable pulmonary *Pseudomonas aeruginosa* infection. Loss of functional CFTR expression is thought to disturb the balance between fluid secretion and absorption into the epithelial layer, leading to net volume depletion of mucus, increased viscosity, and ineffective bacterial clearance. Bacterial infection in turn induces an increased inflammatory response and signaling, thus fueling a vicious cycle of mucus retention, infection, and inflammation.

Mounting evidences indicate that a constitutive inflammatory condition characterizes CF airways regardless of bacterial exposure. CFTR dysfunction results in constitutive, elevated NF-κB activation resulting in increased production of the pro-inflammatory chemokine, interleukin-8 (Vij et al., 2009; Belcher and Vij, 2010; Bodas and Vij, 2010; Hunter et al., 2010). Moreover, the lack of functional CFTR in macrophages has been reported to increase their responsiveness to inflammatory stimuli via uncontrolled TLR4 signaling (Bruscia et al., 2009, 2011) and to affect their capacity to kill *Pseudomonas aeruginosa* (Di et al., 2006; Deriy et al., 2009; Zhang et al., 2010; Del Porto et al., 2011). These findings support the role of CFTR dysfunction in favoring bronchopulmonary inflammation.

Advances in CF treatment have increased the median predicted survival age from less than 5 years in the 1940s to over 37 years presently (Davis, 2006). In addition to therapeutic approaches that target cellular events downstream of the CFTR defect (Mozzillo et al., 2009; Anderson, 2010; Belcher and Vij, 2010; Ratjen and Grasemann, 2012), other strategies focused on the basic CFTR defect have emerged (Riordan, 2008; Sloane and Rowe, 2010; Amaral, 2011; Lukacs and Verkman, 2012). To date, gene therapy has failed to demonstrate a clinical benefit for CF (Riordan, 2008; Amaral, 2011). Thus, pharmacological strategies aimed at correcting mutation-specific CFTR defects (CFTR-repairing therapies) have gained a prominent role in CF drug discovery.

The still partially functional F508del-CFTR protein can be rescued at the PM by means of experimental low thermal conditions (Denning et al., 1992), as well as by so-called correctors, which are molecules that avoid the intracellular retention and degradation of F508del-CFTR protein (Pedemonte et al., 2005; Verkman et al., 2006; Verkman and Galietta, 2009), as extensively reviewed by Molinski et al. and Pedemonte et al. in other chapters of this Special Topic. A number of CFTR corrector molecules have been identified by high-through put screening (Galietta et al., 2001; Pedemonte et al., 2005; Van Goor et al., 2006, 2011). Several CFTR correctors have proved their efficacy in rescuing F508del-CFTR *in vitro*. However, their efficacy in ameliorating the CF lung phenotype, either in pre-clinical models or in CF patients, has not yet established. A recent clinical trial with the most promising CFTR corrector, VX-809 (Van Goor et al., 2011), in F508del-CFTR homozygous patients demonstrated modest dose-dependent reductions in sweat chloride (Clancy et al., 2012). However, beyond this laboratory parameter, no improvement in lung function or CF complications was reported (Clancy et al., 2012; Elborn, 2012).

The pool of F508del-CFTR molecules that can reach the PM after treatment with currently available corrector molecules is unstable. This instability can be explained by carboxyl-terminus heat shock cognate 70 (HSP70)–interacting protein (CHIP)-mediated Ubiquitination of F508del-CFTR (Okiyoneda et al., 2010), followed by redirection of the protein from endosomal recycling toward lysosomal delivery and subsequent degradation (Sharma et al., 2004; Okiyoneda et al., 2010). This seminal observation of Lukacs’ group can explain why CF patients carrying the misfolded F508del-CFTR respond poorly to molecules that increase the activity of CFTR channel (CFTR potentiator) (Davis, 2011; Ramsey et al., 2011). Indeed, the rescued F508del-CFTR is no longer available at the PM for the action of CFTR potentiators. Therefore, combining CFTR correctors and potentiators may be a suitable approach for F508del-CFTR patients, provided that the corrector molecules are effective in increasing F508del-CFTR PM stability after rescue. Currently, phase II clinical studies evaluating the combination of VX-809 and the potentiator VX-770 in CF patients that express F508del-CFTR are underway (Elborn, 2012).

Restoration of a functional proteostasis network by the administration of proteostasis regulators (PRs) has emerged as a novel approach to correct protein misfolding in conformational diseases (Mu et al., 2008; Powers et al., 2009; Gidalevitz et al., 2010; Balch et al., 2011). Therefore, strategies aiming at manipulating peripheral proteostasis could represent a promising area of research in CF drug discovery. Understanding the mechanisms underlying the derangement of proteostasis consequent to defective CFTR function could help improving the search of new drug candidates for CF patients carrying F508del-CFTR mutants.

## Three to Tango in Cystic Fibrosis: CFTR, Transglutaminase 2, and Autophagy

### Defective CFTR function perturbs the post-translational network of CF epithelial cells

An impressive derangement of cellular homeostasis takes place in CF airways. Tissue transglutaminase (TG2) is upregulated in CF epithelial cells at the transcriptional and even more at the post-transcriptional levels (Maiuri et al., 2008). TG2 is a versatile multifunctional protein that changes its function depending on external and internal signals (Nurminskaya and Belkin, 2012). In the presence of high Ca^2+^ levels, TG2 works as a crosslinking enzyme, catalyzing several post-translational modifications of target proteins. At low Ca^2+^ concentrations, TG2 may function as a G-protein or as a protein disulfide isomerase, thus contributing to the functionality of mitochondrial respiratory chain complexes (Nurminskaya and Belkin, 2012). Increased levels of TG2 are observed in several human pathologies including neurodegenerative diseases such as Alzheimer’s, Huntington’s, and Parkinson’s diseases, as well as in chronic inflammatory conditions (Taylor et al., 2003; Malorni et al., 2008; Iismaa et al., 2009; Mastrobernardino and Piacentini, 2010). Most proteins involved in the pathogenesis of neurodegenerative diseases, as huntingtin, ataxin1, tau, and alpha-synuclein, were reported to be TG2 substrates (Mastrobernardino and Piacentini, 2010). Increased TG2 expression has also been reported for glioblastomas, malignant melanomas, and pancreatic ductal adenocarcinomas. Moreover, TG2 expression is often associated with an increased metastatic activity or acquisition of drug-resistance (Antonyak et al., 2004; Karin and Greten, 2005; Kim et al., 2006; Satpathy et al., 2007).

In CF airway epithelial cells, TG2 undergoes small ubiquitin like-modifier (SUMO)ylation (Luciani et al., 2009), a post-translational modification that affects the stability and functions of proteins. SUMOylation is a key player of the post-translational network as it regulates transcription, nuclear translocation, stress responses, and chromatin structure. Moreover, it influences intracellular localization and stability of modified proteins (Geiss-Friedlander and Melchior, 2007; Meulmeester and Melchior, 2008; Tempè et al., 2008). SUMOylation is accomplished by an enzymatic cascade that involves E3 ligases which orchestrate SUMO-modifications in response to stress. We discovered that the protein inhibitor of activated STAT (PIAS)y, which is induced by reactive oxygen species (ROS) and participates in the SUMOylation of NF-κB essential modulator (NEMO) upon genotoxic stress (Mabb and Wuerzberger-Davis, 2006), is upregulated in CF epithelia in response to oxidative stress and then mediates SUMOylation of TG2 (Luciani et al., 2009). Indeed, TG2 contains three SUMO acceptor sites (consensus sequence: ψ_kxE) in its sequence. SUMOylation of lysines is incompatible with the Ubiquitination of these residues (Muller and Hoege, 2001). Thus, TG2 SUMOylation ultimately results in the inhibition of TG2 ubiquitination, thereby preventing its proteasomal degradation. This sustains high intracellular TG2 protein levels, coupled to prolonged TG2 enzyme activation as the result of the elevated ${\text{Ca}}_{i}^{2+}$ content. Indeed, emerging evidence support the role of elevated intracellular calcium concentration in mediating the signaling events that impair homeostasis in CF epithelia, as reviewed by Antigny et al. (2011a) in another chapter of this Special Topic. Although the mechanisms underlying the disturbed calcium homeostasis observed in CF remain incompletely understood, recent studies suggest that impaired calcium signaling may be the result of either increased agonist-mediated activation of G-protein-coupled receptors or abnormal regulation of calcium storage compartments (Egan et al., 2002, 2004; Ribeiro et al., 2005a,b; Norez et al., 2006a,b; Martino et al., 2009). Moreover, the abnormal Ca^2+^ response observed in CF cells depends on the presence of CFTR at the cell surface and this reciprocal regulation of CFTR and Ca^2+^ channels has been described in the literature (Antigny et al., 2011b).

Therefore, in CF, increased intracellular levels of ROS, induced by defective CFTR function, lead to the upregulation of the SUMO E3-ligase PIASy, which facilitates TG2 SUMOylation, persistent high TG2 protein levels, and sustained TG2 activation as the result of “permissive” elevated Ca^2+^ levels. The presence of high TG2 levels might in turn sustain ROS, as it is known that TG2 may stimulate the activity of the mitochondrial respiratory chains (Malorni et al., 2008). Remarkably, inhibiting CFTR, either by gene silencing or by means of pharmacological inhibitors, recapitulates these post-translational modifications of TG2 through upregulating ROS levels in cell lines expressing wild-type (wt)-CFTR (Luciani et al., 2009).

**Figure 1:**
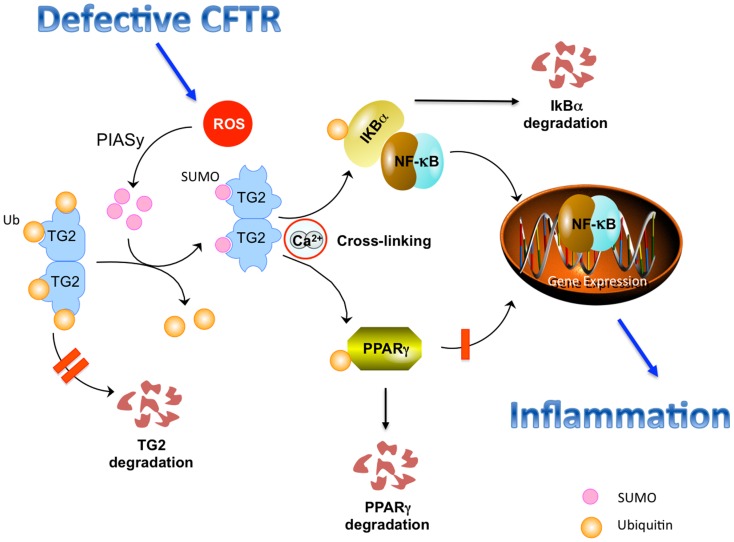
**Defective CFTR-induced perturbation of the post-translational network in CF epithelial cells**. Defective CFTR leads to increased levels of reactive oxygen species (ROS) that increase the levels of the SUMO E3-ligase PIASy, causing TG2 SUMOylation, that, in turn, inhibits TG2 ubiquitination, and avoids its proteosomal degradation, thus sustaining increased TG2 protein levels. Sustained TG2 activation mediates crosslinking of PPARγ and IκBα, which undergo ubiquitination and proteasome degradation. This inhibits nuclear translocation of PPARγ and favors nuclear translocation of NF-κB, stimulating inflammation.

These post-translational changes of TG2 protein, induced by defective CFTR, may have functional implications in epithelial homeostasis. Sustained TG2 activation leads to crosslinking, increased ubiquitination, and functional sequestration of the TG2 substrates peroxisome proliferator-activated receptor (PPAR)γ and IκBα (Daynes and Jones, 2002; Kim et al., 2006; Maiuri et al., 2008). Indeed, the anti-inflammatory molecule PPARγ undergoes SUMOylation in response to its agonists, thus interacting with the N-CoR-histone deacetylase (HDAC) 3 co-repressor complex to maintain a repressor condition (Pascual et al., 2005). TG2-mediated ubiquitination of PPARγ inhibits its SUMOylation and interaction with N-CoR. Similarly, crosslinking and ubiquitination of IκBα inhibits IκBα SUMOylation and favors NF-κB activation and nuclear translocation (Luciani et al., 2009). Both events were reported to favor inflammation in CF airways. Therefore, TG2 can function as a rheostat of the post-translational network in response to CF-associated oxidative stress. TG2 SUMOylation with sustained TG2 activation switches off the post-translational regulatory mechanisms and perturbs the intracellular environment (Figure 1).

**Figure 2:**
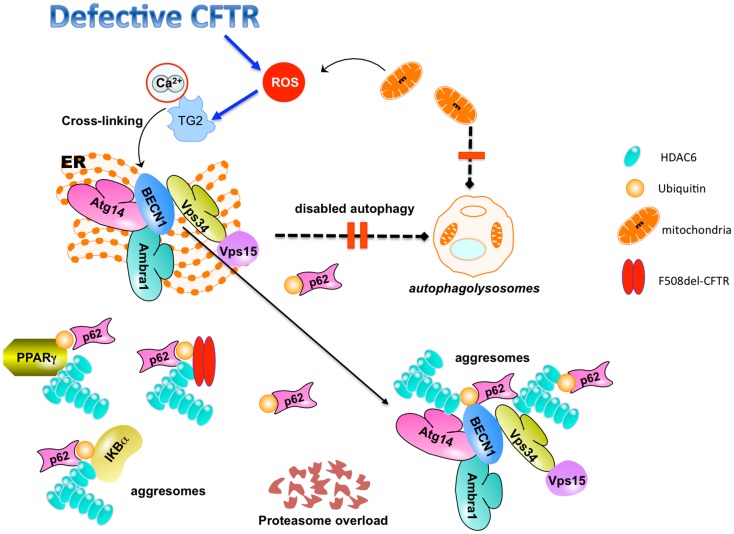
**TG2-mediated inhibition of autophagy in CF epithelial cells**. Defective CFTR-mediated TG2 activation leads to BECN1 crosslinking and displaces BECN1 interactome away from the endoplasmic reticulum (ER). This mislocalization inhibits autophagosome formation, disables autophagy, and induces accumulation of SQSTM1 (p62). SQSTM1 accumulation leads to proteasome overload and favors sequestration of cross-linked TG2 substrates (PPARγ, IκBα, BECN1) within HDAC6^+^ aggresomes. The combined inhibition of protein and aggresome turnover may also favor the accumulation of F508del-CFTR (together with SQSTM1) within HDAC6^+^/ubiquitin^+^ intracellular aggregates. Defective autophagy inhibits the clearance of damaged mitochondria that contribute to the generation of pro-inflammatory ROS.

TG2-mediated protein ubiquitination and crosslinking may lead to protein aggregation and proteasome overload, thus favoring aggresome formation (Muma, 2007; Dohm et al., 2008). Misfolded or post-translationally modified proteins that cannot be degraded by the proteasome machinery can be stocked in the cytoplasm in the form of aggresomes (Kawaguchi et al., 2003; Kirkin et al., 2009). Accordingly, ubiquitylated PPARγ and IκBα aggregates are sequestered within histone-deacetylase (HDAC)6^+^/vimentin^+^ intracellular aggresomes in CF epithelial cells (Figure 2).

Therefore, proteostasis of F508del-CFTR epithelia is affected by a combination of genetic defect (resulting from the misfolded CFTR protein) and post-translational alterations (through the ROS/TG2 axis).

TG2 is localized in multiple cellular compartments including cell surface and extracellular matrix. Besides its crosslinking activity on ECM substrates, extracellular TG2 is also endowed with PDI, or GTPase functions (Nurminskaya and Belkin, 2012). However, the potential relevance of the extracellular TG2 in CF is still unknown.

### Defective CFTR disables autophagy

Given the overproduction of ROS together with the endoplasmic reticulum (ER) stress induced by the mutant CFTR, one would expect an activation of autophagy in F508del-CFTR homozygous epithelial cells. Autophagy is pivotal in promoting cellular clearance of protein aggregates and removal of ROS sources, such as damaged mitochondria (Mizushima et al., 2008; Kirkin et al., 2009; Korolchuk et al., 2009; Kroemer et al., 2010; Moreau et al., 2010). Surprisingly, however, human and mouse CF airways exhibit a pronounced defect in autophagy, as indicated by reduced autophagosome formation, and the accumulation of sequestosome 1 (SQSTM1), a major autophagic substrate also known as p62. This occurs in spite of the normal expression of major autophagy genes (Luciani et al., 2010, 2011). A defective autophagic response to bacterial infection has also been reported in murine CF macrophages. Reduced autophagosome formation in CF macrophages promotes *Burkholderia cenocepacia* survival and hypersecretion of IL-1β (Abdulrahman et al., 2011).

Autophagy results in the lysosomal degradation of cytoplasmic organelles or cytosolic components after their sequestration in two-membraned vesicles (Kroemer et al., 2010; Yang and Klionsky, 2010; Codogno et al., 2011; Mizushima et al., 2011). In the last few years, autophagy has emerged not just as a simply degradative process, but also as a cellular mechanism essential for the maintenance of cellular homeostasis and of the energetic balance (Kroemer et al., 2010). Thus, disabled autophagy is associated with and is relevant to several human diseases including cancer, viral infection, neurodegenerative diseases, respiratory pathologies, and chronic inflammatory disease (Levine et al., 2011; Rubinsztein et al., 2011; Sridhar et al., 2012; Patel et al., 2013).

Through which mechanisms is autophagy inhibited in CF? We have demonstrated that the inhibition of autophagy in CF epithelial cells is part of the complex perturbation of the post-translational network consequent to defective CFTR function. Disabled autophagy in CF epithelial cells is a consequence of TG2-mediated crosslinking and functional sequestration of BECN1, a major player of autophagosome formation, which exhibits target sites (QP, QxxP) for crosslinking by TG2 (Luciani et al., 2010).

BECN1 is a haploinsufficient tumor suppressor protein that is essential for autophagy (Sinha and Levine, 2008; He and Levine, 2010; Maiuri et al., 2010). Accumulating evidence indicate that BECN1 dissociates from Bcl-2 during stress conditions, such as starvation, thus promoting autophagy (Pattingre et al., 2005; Maiuri et al., 2007, 2010; Axe et al., 2008; Hayashi-Nishino et al., 2009). Subsequently, BECN1 interacts with the class III phosphatidyl-inositol 3 kinase (PI3K), human vacuolar protein sorting (hVps)34 (Matsunaga et al., 2009; Zhong et al., 2009), facilitating its activation. The ER-associated class III PI3K activity is crucial for the initiation of autophagosome formation (Axe et al., 2008; Hayashi-Nishino et al., 2009).

Reduced BECN1/Bcl-2 interaction upon starvation is observed in CF cells, suggesting an intracellular environment favorable to autophagy induction. Moreover, BECN1 interacts with the essential components of the PI3K complex IIIhVps34, hVps15, Ambra1, as well as with Atg14L, a BECN1 interactor that diverts hVps/Class III PI3K into an autophagic role (Liang et al., 2008; Matsunaga et al., 2009). However, in CF epithelial cells, the BECN1 interactome is dislodged away from the ER as a consequence of BECN1 crosslinking and is sequestered within HDAC6^+^ aggresomes. This impairs autophagosome formation in CF cells (Luciani et al., 2010).

Autophagy deficient CF cells accumulate SQSTM1 (p62), an ubiquitin-binding (and LC3-binding) protein (Bjørkøy et al., 2005; Kirkin et al., 2009; Mathew et al., 2009; Duran et al., 2011) that is selectively degraded by autophagy. Autophagy upregulation has been reported as a compensatory response to proteasome inhibition, thus revealing a crosstalk between the proteasome-based and the autophagy-based degradation pathways (Komatsu et al., 2007; Kirkin et al., 2009; Korolchuk et al., 2009; Lamark and Johansen, 2010). SQSTM1 accumulation resulting from autophagy inhibition contributes to proteasome overload and favors aggresome formation, while disabled autophagy inhibits the clearance of such protein aggregates. Altogether, the combined inhibition of protein and aggresome turnover may also influence the fate of misfolded CFTR. Indeed, the enforced expression of F508del-CFTR in CF epithelial cell lines favors the accumulation of misfolded CFTR (together with SQSTM1) within HDAC6^+^/ubiquitin^+^ intracellular aggregates (Luciani et al., 2010).

We suggest that this cascade of events can generate a vicious feed-forward loop, as it impairs the clearance of damaged mitochondria, thus increasing ROS generation that in turn enhances TG2 activation and BECN1 sequestration, further sustaining airway inflammation (Figure 2).

### Restoring proteostasis ameliorates lung inflammation through rescuing autophagy in CF

Inhibiting TG2 activity by cystamine (and its reduced form cysteamine) or targeting ROS can reduce inflammation in F508del-CFTR airways, both *in vivo* in F508del-CFTR homozygous mice (*Cftr*^F508del^ mice) and in *ex vivo* using explanted human polyp biopsies from CF patients (Raia et al., 2005; Luciani et al., 2010). The effects of cystamine on airway inflammation are mediated by its ability to rescue BECN1 and autophagy, as either BECN1 depletion used *in vitro* or administration of the PI3K complex III inhibitor 3-methyl-adenine (3-MA) used *in vivo* in *Cftr*^F508del^ mice, abrogated these beneficial effects of cystamine. Either enforced BECN1 overexpression or SQSTM1 depletion *in vivo* recapitulated the effects of cystamine in ameliorating lung inflammation in *Cftr*^F508del^ mice (Luciani et al., 2010, 2012). Similarly, cysteamine has already been successfully used in mouse models of Huntington’s disease to improve disease-related phenotype (Karpuj et al., 2002).

Importantly, we demonstrated that amelioration of lung inflammation in *Cftr*^F508del^ mice secondary to cystamine treatment persists up to 10 days beyond cystamine withdrawal, unless the rescue of BECN1 and autophagy are inhibited by the administration of 3-MA during washout (Luciani et al., 2012). These data suggest the provocative hypothesis that, once the cellular environment has been re-directed toward a physiological status, a driving force is re-established within the cell, so as to prolong these beneficial effects. Could this “newly re-established” player be the functional CFTR itself at the epithelial surface? Indeed, manipulating proteostasis might actually improve the function of misfolded proteins (Balch et al., 2008; Roth and Balch, 2011).

## Targeting Autophagy as a New Strategy to Enable the Action of CFTR Potentiators on F508del-CFTR

F508del-CFTR rescued at the PM by means of corrector strategies is rapidly dismissed and re-directed to lysosomes for degradation (Sharma et al., 2004; Okiyoneda et al., 2010, 2011; Lukacs and Verkman, 2012). Accordingly, the biochemical half-life of PM F508del-CFTR is lower than 4 h (Lukacs et al., 1993; Heda et al., 2001). Therefore, F508del-CFTR is no longer present at the PM and cannot interact with CFTR potentiators after rescue.

Recently, we have reported that overexpression of BECN1, administration of cystamine, or depletion of SQSTM1 by RNA interference, can favor the trafficking of F508del-CFTR protein to the epithelial cell surface *in vitro* in CF epithelial cell lines (CFBE41o- or IB3-1, carrying F508del/F508del or F508del/W1282X CFTR, respectively), *ex vivo* in nasal polyp biopsies from CF patients, and *in vivo* in *Cftr*^F508del^ mice. Interestingly, these treatments can restore a functional CFTR in CF cell lines and in primary brushed nasal epithelial cells from F508del-CFTR homozygous patients (Luciani et al., 2012).

Therefore, PR-based strategies in CF (as administration of cystamine or genetic restoration of BECN1) may have a dual effect, as they reduce lung inflammation while rescuing a functional mutant CFTR to the epithelial surface.

### Targeting autophagy improves F508del-CFTR PM stability in airway CF epithelia well beyond drug washout

In addition to its ability to rescue F508del-CFTR, cystamine is effective in delaying the disposal of PM resident F508del-CFTR protein and generates permissive conditions to prolong F508del-CFTR PM residence well beyond cystamine washout. These effects are mediated by the ability to restore BECN1 and autophagy, as both BECN1 depletion and 3-MA abrogate the beneficial effects of cystamine. PM resident mutant F508del-CFTR is still functional after cystamine withdrawal, as it retains the ability to respond to forskolin added together with CFTR potentiators (as genistein or VX-532 or VX-770) well beyond the washout period. This prolonged function of F508del-CFTR was observed in CF cell lines, as well as in primary brushed nasal epithelial cells from F508del-CFTR homozygous patients. Moreover, cystamine sustains F508del-CFTR re-location at the lung epithelial surface after 10 days following washout *in vivo* in *Cftr*^F508del^ mice, unless that cystamine was combined with 3-MA (Luciani et al., 2012).

These effects of cystamine can explain how the anti-inflammatory effects as a result of the restoration of autophagy (either via pharmacological intervention, as cystamine, or by the enforced expression of BECN1) persist well beyond its withdrawal in *Cftr*^F508del^ mice. They probably rely on CFTR itself. Indeed, the pro-autophagic effects of cystamine persist after 10 days following cystamine withdrawal unless CFTR was depleted during washout, suggesting that these anti-inflammatory effects are mediated by the ability to sustain a functional CFTR at the cell surface (Luciani et al., 2012). Therefore, sustained CFTR function at the PM can interrupt the cascade of ROS generation, TG2 activation, BECN1 sequestration, and autophagy inhibition, and ultimately reduce lung inflammation.

These findings could also explain how cystamine, which is not an autophagy inducer, is highly effective in restoring autophagy within a “*CF environment*.” The fact that cystamine can regulate peripheral proteostasis is also supported by the observation that cystamine (but not CFTR correctors as VX-325 or Corr-4a) is effective in sustaining PM stability of F508del-CFTR even if it has previously been rescued at the PM by low temperature.

### Targeting autophagy enables the beneficial action of potentiators on F508del-CFTR

These findings indicate that PRs may be used to rescue and stabilize F508del-CFTR at the PM of CF epithelial cells. In principle, this strategy could lower the ER quality control (QC) threshold of all misfolded proteins, thus interfering with the QC fidelity. However, besides its effects on F508del-CFTR rescue at the PM, cystamine can also delay the disposal of PM resident F508del-CFTR protein. The evidence discussed in this review supports that defective CFTR suppresses autophagy within the CF epithelial environment (though the ROS/TG2 pathway), and, conversely, that rescuing autophagy can restore a functional CFTR at the PM. Altogether, these insights suggest the existence of a vicious cycle in which defective CFTR functions destabilizes the CFTR protein and that can be interrupted by cystamine.

Considering these factors, one would expect that sustaining PM residence of F508del-CFTR by PRs, could allow potentiators to improve Cl^−^ transport though PM resident CFTR molecules. Indeed, our recent data indicate that, if proteostasis has been previously restored by cystamine, potentiators can become effective in sustaining the anti-inflammatory effects of cystamine *in vivo* in *Cftr*^F508del^ mice. Importantly, genistein, which has no effects on its own in *Cftr*^F508del^ mice, synergistically interacts with cystamine to reduce lung inflammation triggered by the challenge with lipopolysaccharide (LPS) from *Pseudomonas aeruginosa*. The same effects are observed if proteostasis has been previously re-established in *Cftr*^F508del^ mice by means of genetic manipulations, such as lentiviral expression of BECN1 or shRNA-mediated depletion of SQSTM1 (Luciani et al., 2012).

These observations indicate that pharmacological measures that ameliorate the cellular environment in which mutant CFTR traffics, instead of specifically targeting the misfolded protein itself, can result in improved disease outcome.

## Perspectives

### CFTR-repairing strategies and anti-inflammatory therapies: Two sides of the same coin?

The data discussed in this review suggest that different approaches could be envisaged to control CF lung inflammation. Some therapies focus on conventional or emerging anti-inflammatory molecules, downstream of CFTR. Others, as cystamine and other yet-to-be-developed PRs, rely on the rescue and stabilization of functional CFTR at the PM. Apparently, cystamine can interrupt the dangerous cycle leading to lung inflammation, thus opening a new scenario in the search of the most appropriate CFTR-repairing strategy.

Searching the appropriate CFTR corrector is a challenging issue in drug development. An ideal drug candidate for the treatment of F508del-CFTR patients should not only aim at rescuing trafficking of mutant CFTR, be it through the conventional Golgi-mediated exocytic pathway (Ward et al., 1995; Quinton, 1999; Amaral, 2004, 2011) or the unconventional GRASP-dependent secretory pathway (Gee et al., 2011), but also at sustaining the rescued mutant CFTR at the PM, to allow the combined action of potentiators on PM resident F508del-CFTR. Ideally, one single molecule should be endowed with all these properties to minimize undesirable effects. Our recent observations suggest that F508del-CFTR patients could be sequentially treated with two single pharmacological agents, first with cystamine and then with CFTR potentiators.

The findings discussed in this review also highlight the importance of testing F508del-CFTR correctors for their capacity to exert a prolonged control of lung inflammation in pre-clinical models, before initiating clinical trials. So far, our strategy has been successfully tested in nasal polyp biopsies from F508del-CFTR homozygous patients as well as *in vivo* in F508del-CFTR homozygous mice (Luciani et al., 2012). At this stage, clinical trials on CF patients are justified.

## Conflict of Interest Statement

The authors declare that the research was conducted in the absence of any commercial or financial relationships that could be construed as a potential conflict of interest.
